# A Comparative Study on Hygric Properties and Compressive Strength of Ceramic Bricks

**DOI:** 10.3390/ma15217820

**Published:** 2022-11-05

**Authors:** Piotr Stępień, Edyta Spychał, Karol Skowera

**Affiliations:** Faculty of Civil Engineering and Architecture, Kielce University of Technology, Al. Tysiąclecia Państwa Polskiego 7, 25-314 Kielce, Poland

**Keywords:** ceramic bricks, compressive strength, water absorption, capillary rise, micro-CT, masonry wall, MIP

## Abstract

This article analyzes the results of capillary rise, compressive strength and water absorption tests on solid ceramic bricks from existing structures and demolition materials taken from 11 different structures. In addition (for more extensive interpretation and evaluation of porosity), tests were performed for the selected series of bricks using a mercury porosimeter (MIP) and a micro computed tomography (micro-CT). Contemporary bricks (2 series) were also evaluated for comparison purposes. The conducted tests indicate that bricks obtained from different sources are characterized by “individual” relation of compressive strength and porosity, and “individual” relation of water absorption coefficient and porosity. In addition, on the basis of the results obtained in the study, compared with the literature data, it can be deduced that ceramic bricks with a water absorption coefficient of less than 50 g/m^2^s^0.5^ are characterized by a compressive strength of more than 80 MPa. As the research shows, the properties of bricks even from a single building can differ one from another, which can result in varying durability even within a single building. When choosing a material during the renovation or restoration of facilities, it is important to perform tests on the physical and mechanical properties of the original material, which will be reused.

## 1. Introduction

Moisture is one of the main factors affecting the durability of masonry structures [1,2,3]. The amount of water with which the material is in contact directly determines the degree of damage associated with cyclic freezing/thawing, salt attack and biological corrosion. The appearance of water in masonry not only affects durability but also causes deterioration of thermal performance, as well as mechanical properties of the masonry [4,5]. When there is no damp proofing or when the damp proofing no longer serves its purpose, water is easily able to penetrate the pore space. As a result of capillary forces, water can be transported in the masonry up to a height of several meters. This is due to the fact that the pores in ceramics create one continuous system. Significant amount of water on the structure can appear, not only by the capillary rise but also due to wind-driven rain [6]. In the case of buildings, of which solid brick is the main structural element, significant deterioration can take place over hundreds of years, and in extreme cases, several years. This will depend on the amount of water acting on the structure [7]. Another factor determining the durability of masonry is its water absorption, which is directly related to porosity. These product properties are determined at the production stage. The main factors affecting durability, pore size distribution and porosity are the mineral composition, moisture content of the raw material mass, production technology, the thermal curve of firing and, most importantly, the maximum firing temperature [8,9,10,11]. As the firing temperature increases from 900 °C to 1100 °C, the pore size distribution gradually changes [8,9,10], the proportion of pores below 1 μm decreases, and at the same time the open porosity decreases [7,12]. What can be observed in structures as uneven dampness of masonry, reducing the durability of the structure [13,14], can also reduce the thermal insulation properties of the building [15]. In addition, absorption decreases, and compressive and flexural strength increases [16]. As Maage and Šveda [9,12] show, as the proportion of pores above 3 μm increases, the resistance of ceramic bricks to cyclic freezing-thawing of water increases. The presence of pores above 3 μm has a beneficial effect on reducing the decrease in compressive strength after freeze-thaw cycles [17].

A factor that can further deteriorate the quality of the product is the presence of carbonates in the mineral composition. At temperatures above 700 °C, the decomposition of calcite and dolomite occurs, accompanied by the formation of cracks (fracturing) and porous structures with porosity greater than that of the surrounding medium [11,18]. When bricks are fired at temperatures above 1050 °C, the influence of carbonates diminishes.

The presented examples show that the properties of clay bricks depend on many factors occurring from the stage of production to the stage of operation of the structure. Determination of preservation and durability state of masonry walls is especially difficult because of the differences in individual bricks properties and allocation in structure. Each part of the wall may be exposed to the environment in a completely different way and the sources of water present in the masonry may be completely different [19,20]. Because water is the main factor influencing deterioration of building materials, hygric properties are drawing a persisting amount of attention. Parameters influencing the hygric properties were widely investigated, e.g., vacuum saturation, capillary absorption, vapor diffusion [21,22,23].

In order to determine the technical condition of masonry structures, tests are carried out on mechanical properties, porosity, capillary rise and the number of salts present in the masonry. The relevance of these parameters on the behavior of the bricks is evidenced by the requirements of the American and Canadian standards given in Table 1. According to the standards ASTM C67-07a [24] and CSA A82-06 [25], ceramic masonry units are considered frost resistant when these meet the criterion of maximum saturation coefficient or maximum cold-water absorption. It is not required to meet both requirements. In addition, ceramic products must have a compressive strength of not less than 17.2 MPa. In the case of bricks obtained from existing buildings, the compressive strength can take values from a few MPa to as high as 80 MPa, depending on the material taken [26,27]. Research by Grubeša et al. [17] and Stryszewska et al. [28] shows that brick samples with strengths greater than 15 MPa can show significant signs of deterioration. On the other hand, it was noted that bricks with the main proportion of pores with a radius greater than 1 μm had good resistance to adverse effects of moisture (no damage on the external surface).

The water absorption coefficient C_1_ (Equation (1), also called capillary absorption coefficient) can be used to compare the rate of water absorption by capillary uptake. According to EN 1925:2001 [29], this parameter is defined as the ratio of the mass of absorbed water, during the capillary rise test, to the product of the contact area of the sample with water and the square of time.

(1) $$C_{1}=\frac{m_{i}-m_{d}}{A\sqrt{t_{i}}}$$

where t_i_ is the time from the beginning of capillary soaking, m_i_ is the mass of sample with water after time t_i_, m_d_ is the mass of dry sample, A is the contact area of the sample with water. 

**Figure 1:**
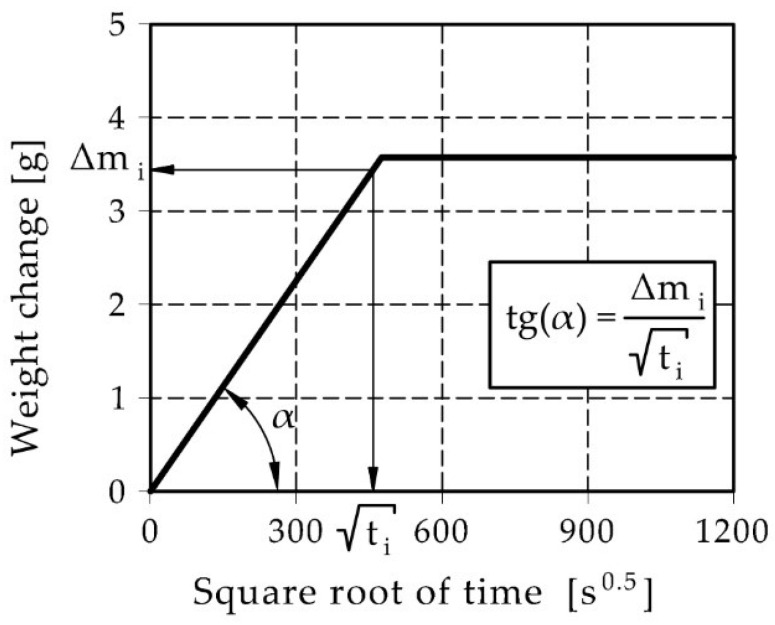
Tangent of slope of the initial section of the sample weight increments.

The physical of the component (m_i_ − m_d_)/t^0.5^ in formula A is shown in Figure 1.

As research [30] shows, in the case of ceramic materials produced by the authors, it is possible to predict their frost resistance on the basis of capillary rise tests. Water absorption coefficient can be used to evaluate water penetration caused by wind-driven rain [31]. 

The examples cited above from the literature on the factors affecting the durability of masonry clearly demonstrate the need to perform tests on the material’s ability to moisture transport, absorption and compressive strength of the masonry element. The problem of assessing the technical condition of existing buildings, in which clay bricks are the main structural element, is all the time relevant [32,33,34,35]. In particular, when materials are sought for the reconstruction of an existing element or when the reuse of post-demolition material is considered [36,37,38,39]. For reconstruction purposes, bricks with well-identified properties should be used. Before application, defects in material not visible on the surface can be detected using nondestructive methods [40,41].

This paper focuses on the issues of variation in parameters related to hygric properties and compressive strength. In the literature, the most common focus has been on capillary rise testing of bricks from a single source. If samples come from different sources, then mechanical properties and porosity tests are performed without performing capillary rise tests. An attempt was made to evaluate the variation in the parameters of solid ceramic bricks taken from different sources. Typically, in articles, research on ceramic materials focuses on determining their physical or mechanical properties, sometimes supplemented by research carried out with the MIP method of pore distribution. In this work, in addition to common standard tests and porosimetry tests, an analysis of the homogeneity and possible defects of bricks using the non-standard micro-CT method was performed. Comparing the results of computer microtomography with the results of capillary rise tests performed for two surfaces of the same sample, it was shown that even an apparently from the outside homogeneous material is characterized by variable behavior in contact with water. This is due to the presence of microcracks, cracks or other defects in the internal structure of the tested bricks, which were confirmed by micro-CT examination.

## 2. Materials and Methods

### 2.1. Samples

Thirteen series of samples of solid ceramic bricks, a total of 66 masonry elements, were prepared for testing. These were bricks obtained from existing buildings, as well as demolition bricks: materials from the 17th (1 series), 18th (1 series), 19th (1 series), 20th (8 series), and two series of new bricks from the 21st century. The number of samples in each series depended on the availability of materials. Different brick series is labelled in Table 2 as follows: N for new bricks from the XXI century, O for bricks from period older than XXI century, number corresponds to different brick series and the century of samples is added. Table 2 summarizes all 13 series of samples, along with the exact number of bricks investigated. The samples were cleaned of dirt before examination, adhering mortar was removed with a brush with metal bristles. No cracks running through more than 20% of the specimen’s thickness were observed on the outer surface of the samples. 

### 2.2. Test Procedure

#### 2.2.1. Samples Preparation

The samples were cleaned of adhering mortar with a wire brush before testing. Solid bricks were cut into 3 cubic specimens of 6 × 6 × 6 cm and one rectangular specimen of 4 × 4 × 16 cm. The volumetric density and porosity of the samples were determined using the vacuum hydrostatic method on the cubic samples. Samples of 4 × 4 × 16 cm were used to test the water absorption coefficient and water absorption after 24 h and 7 days. After the vacuum saturation test, the cubic samples were dried to a constant weight at 45 °C and tested for compressive strength using a hydraulic press. Of the bricks tested, 10 were selected for which pore space tests were performed using X-ray/microcomputed tomography and 5 of them were tested using mercury intrusion porosimetry.

#### 2.2.2. Capillary Rise Test Procedure

Before the test, the samples were dried to a constant weight at 45 °C. They were then cooled in a desiccator to 20 °C. The samples were placed on the grid smaller of the surface so that they were submerged to a depth of 4 mm. The weight of the samples was determined after 5, 10, 15, 30 min, and 1, 2, 3, 6, 24 h, by removing them from the water and wiping the excess from the underside with a damp cloth, and weighing them on a scale to the nearest 0.01 g. The samples on the side surface were not insulated. The weight gain after 24 h and 7 days was determined. Water capillary absorption was calculated using Equation (1) [29].

#### 2.2.3. Procedure for Testing Porosity, Volumetric Density and Vacuum Saturation Using the Vacuum Method

Before testing, the samples were dried to a constant weight at 45 °C. They were then placed in a chamber from which air was removed to a pressure of 0.1 atmosphere. The pressure of 0.1 atmospheres was maintained for 30 min, after which the materials were flooded with distilled water at 80 °C. After the samples were kept in water for three days, their porosity and bulk density were determined by hydrostatic method.

#### 2.2.4. Procedure for Testing Water Absorption

Before the test, 4 × 4 × 16 cm samples were dried to a constant weight at 45 °C. The samples were cooled in a desiccator to 20 °C. The samples were placed in water on washers after which they were flooded with distilled water. Water level was 5 cm above the samples’ top.

#### 2.2.5. Mercury Intrusion Porosimetry

Pore size distribution and porosity was determined using a Micrometric AutoPore IV 9500 mercury intrusion porosimeter. Cylinder samples of about 12 mm in diameter and 10–14 mm in length were prepared separately for the test. Before testing, the samples were dried to a constant weight at 75 °C for 5 days. The samples were then cooled to 20 °C in a desiccator.

#### 2.2.6. Procedure for Micro-CT Examination

The study was performed using a Nikon XT H 225 ST CT scanner. The scan was performed with a rotating target gun (rotating target). Its maximum voltage is 225 kV and power is 450 W. A voltage of 220 kV and an intensity of 432 μA were used to scan the samples. The exposure time was 250 ms. 4476 projections were made. The resolution (voxel size) was 0.039 mm (39 μm). CT PRO 3D software was used for reconstruction, and VG Studio Max 3.4 was used for analysis.

## 3. Results and Their Analysis

**Figure 2:**
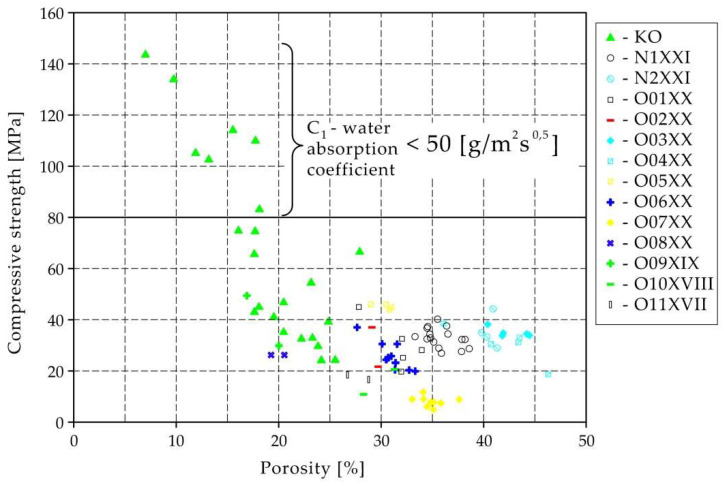
Relationship of compressive strength and porosity.

**Figure 3:**
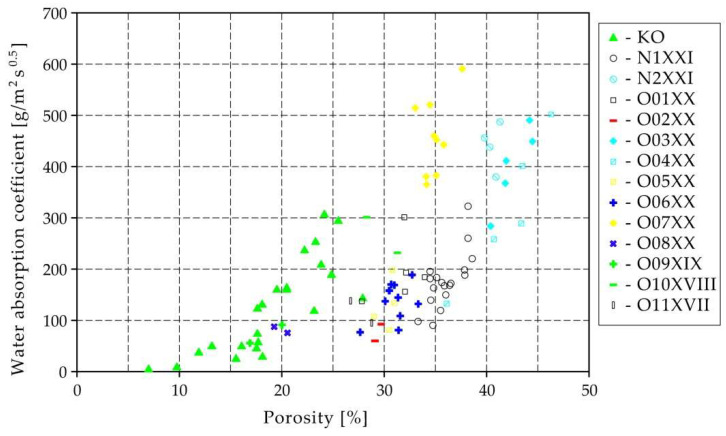
Relationship of water absorption coefficient and porosity.

Figure 2 shows the value of the average compressive strength of each brick. In addition, the results of 9 series of bricks (23 bricks) from Koroth’s doctoral dissertation [42] are plotted in Figure 2 and Figure 3. They are signed as samples of KO.

Figure 3 shows a significant variation in the water absorption coefficient. At a given porosity, the values can vary several times relative to each other. For specimens with compressive strengths above 80 MPa, water absorption coefficient values were below 50 g/m^2^s^0.5^.

**Figure 4:**
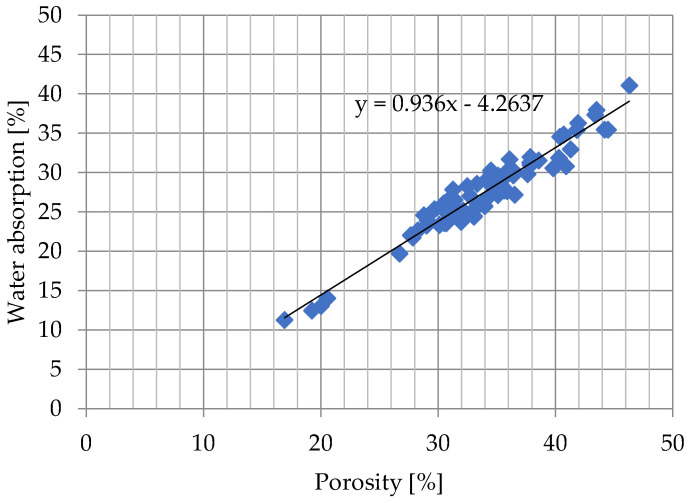
Water absorption after 24 h of submersion in cold water.

**Figure 5:**
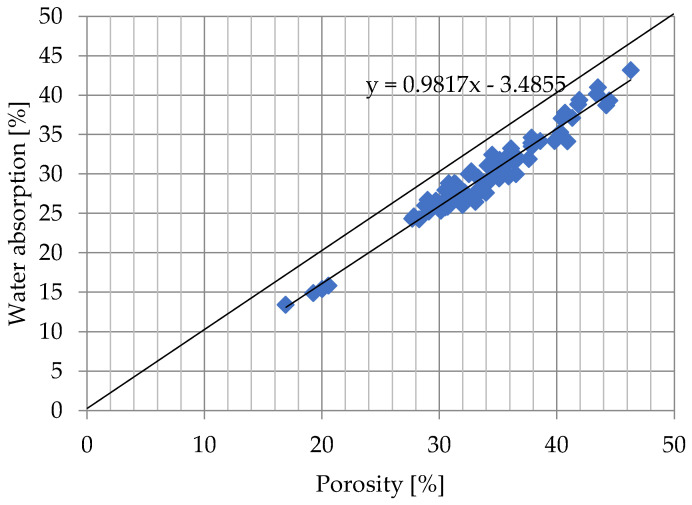
Water absorption after 7 days of submersion in cold water.

Water absorption results for all samples after 24 h and 7 days of soaking were shown in Figure 4 and Figure 5.

After 24 h as well as after 7 days of soaking, there is a similar volume of pores inside the samples in the pore space that are not occupied by water (Figure 4 and Figure 5). This volume does not depend on the porosity of the samples. 

In the case of Figure 4, the fir factor R^2^ equals 0.938, which denotes that this model explained 93.8% of changes in the response value. In case Figure 5 R^2^ equals 0.956, that this model explained 95.6% of changes in the response value. There is a visible relationship between water absorption and porosity of bricks tested. Ceramic bricks with the porosity of about 20% have water absorption of about 15%, whereas ceramic bricks characterized by porosity in the range from 26% to 46% have water absorption in the range of 20% to 45%. Probable relation between percentage of pores not occupied by water to open porosity is responsible that the porosity of 18% is the separating value between frost-resistant samples and those that are not frost-resistant or of questionable frost resistance, which is consistent with the analysis presented in [43]. The volume of pores unoccupied by water after 7 days of ordinary soaking in the tests conducted was about 3.48%, relative to the total volume of the sample. In the case of the Bracka [43] study, this value was equal to 4.9% for solid ceramic bricks.

For the selected series of samples compressive strength and water absorption coefficient trend lines were determined and were shown in Figure 6, Figure 7, Figure 8, Figure 9, Figure 10, Figure 11, Figure 12, Figure 13, Figure 14 and Figure 15.

**Figure 6:**
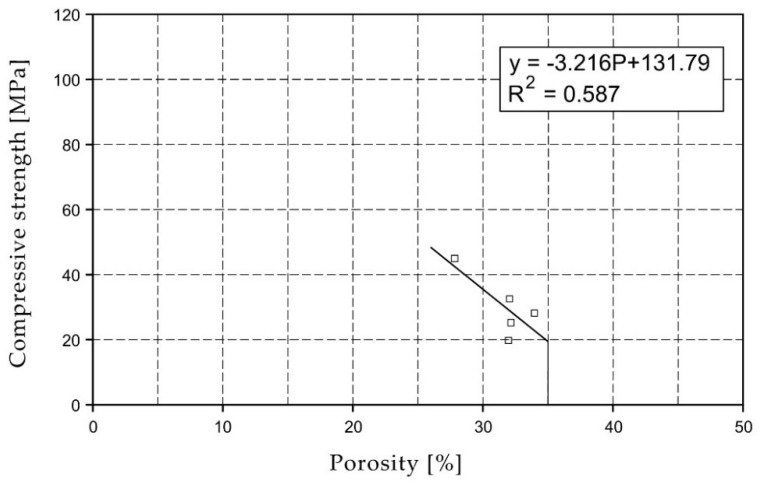
Compressive strength of O01XX series bricks.

**Figure 7:**
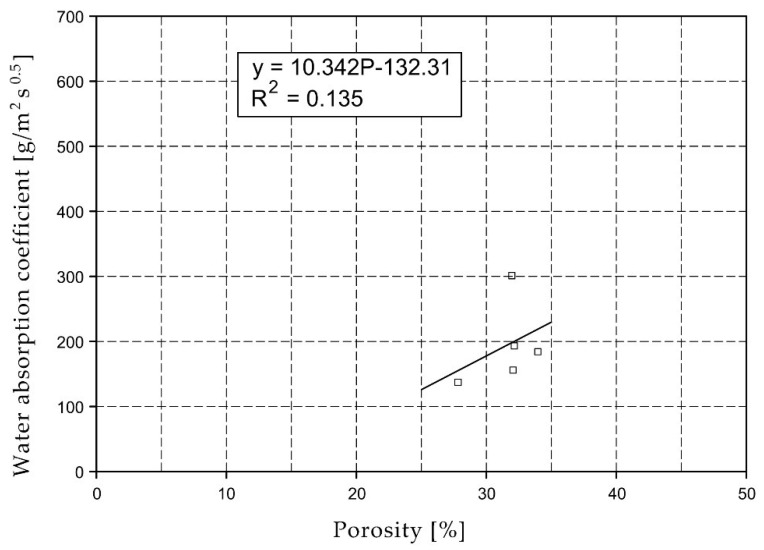
Water absorption coefficient of O01XX series bricks.

**Figure 8:**
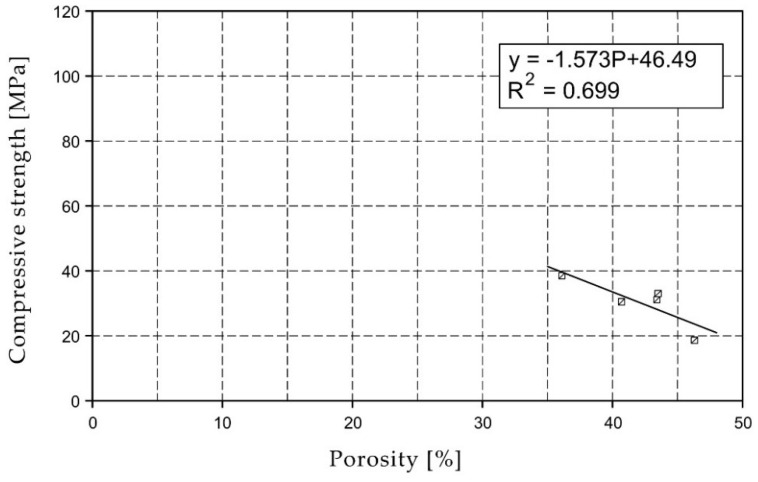
Compressive strength of O04XX series bricks.

**Figure 9:**
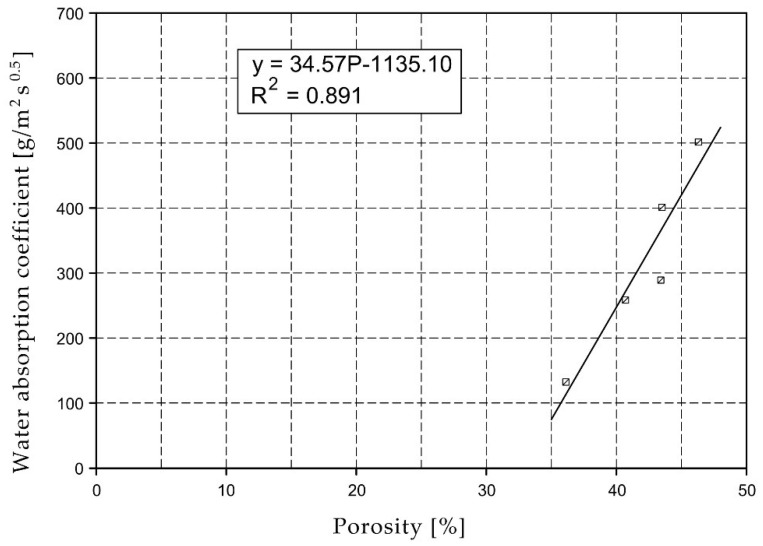
Water absorption coefficient of O04XX series bricks.

**Figure 10:**
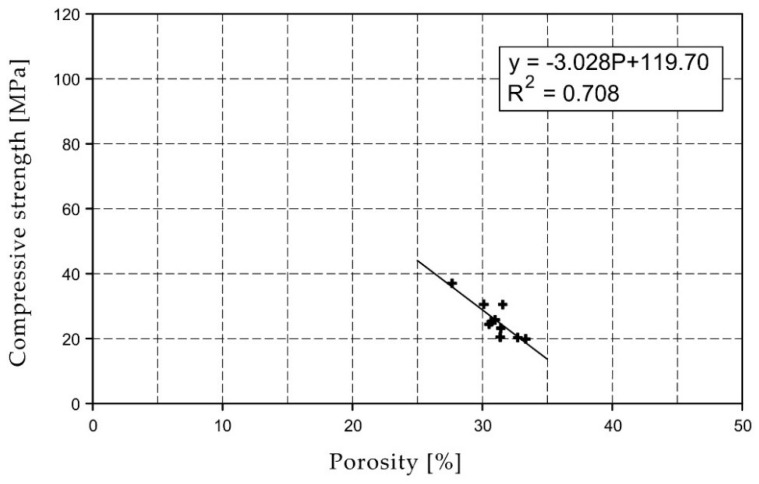
Compressive strength of O06XX series bricks.

**Figure 11:**
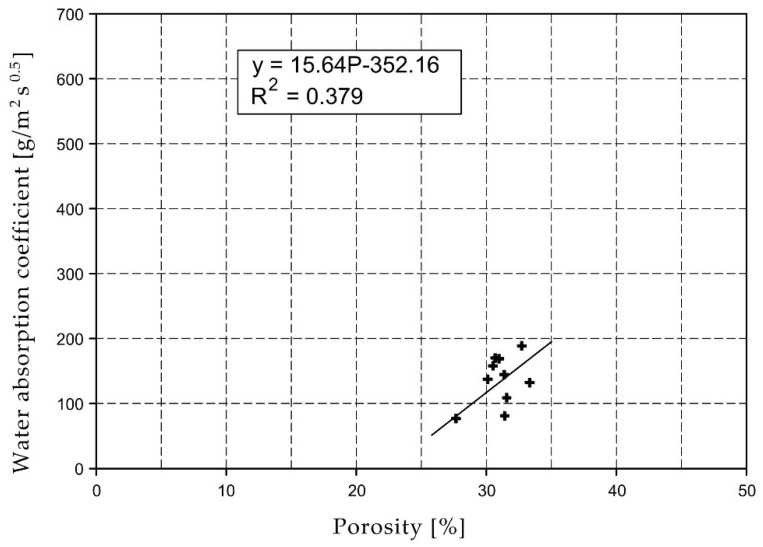
Water absorption coefficient of O06XX series bricks.

**Figure 12:**
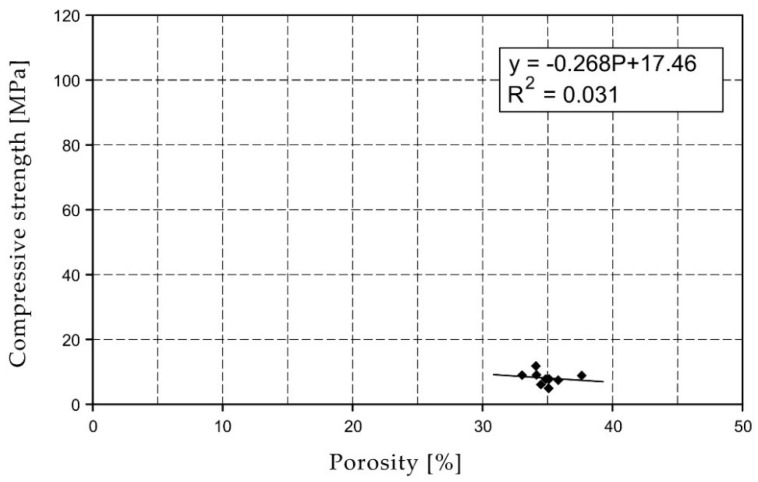
Compressive strength of O07XX series bricks.

**Figure 13:**
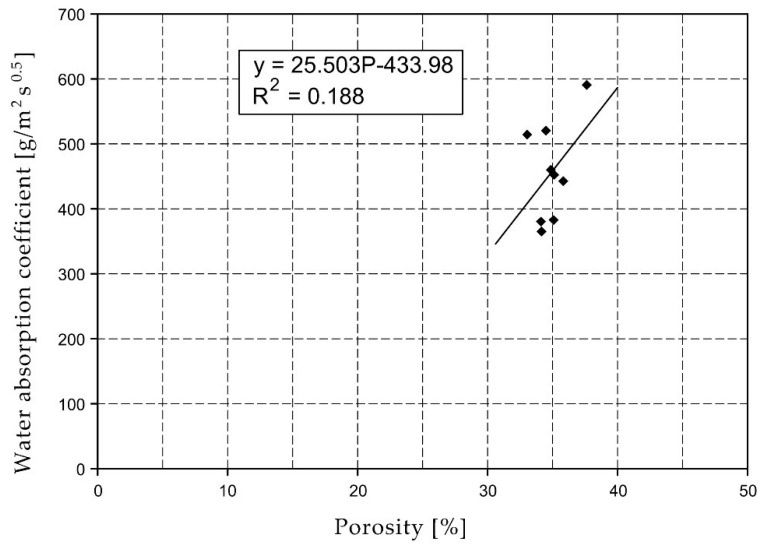
Water absorption coefficient of O07XX series bricks.

**Figure 14:**
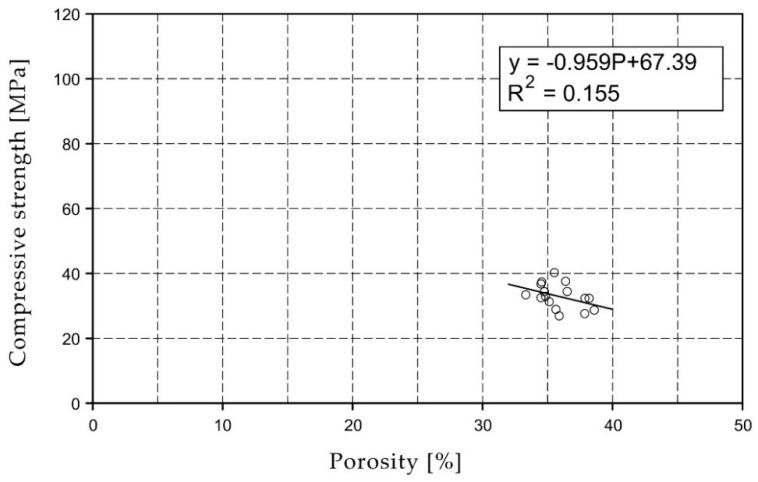
Compressive strength of N01XXI series bricks.

**Figure 15:**
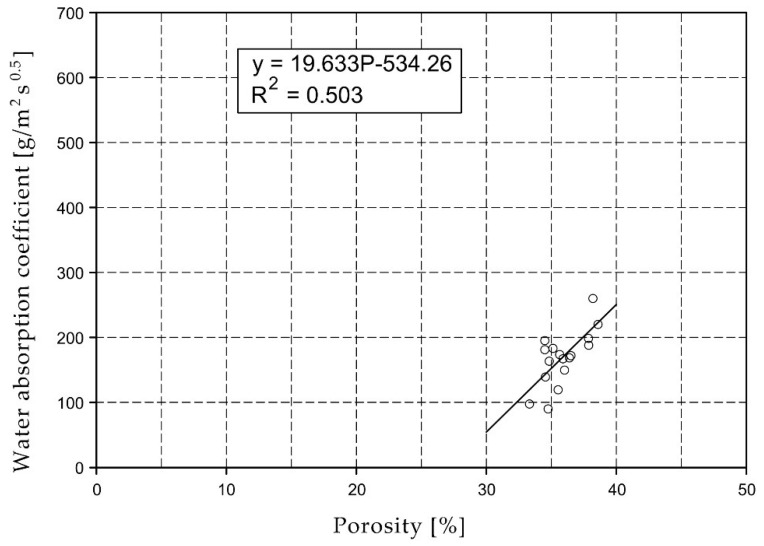
Water absorption coefficient of N01XXI series bricks.

Considering the Koroth [42] results (Figure 2 and Figure 3) and the trend lines in Figure 6, Figure 7, Figure 8, Figure 9, Figure 10, Figure 11, Figure 12, Figure 13, Figure 14 and Figure 15, it can be deduced that each series of bricks is characterized by its own strength-porosity relationship (comparing the test results and the model fit factor for each of the graphs). The standard deviation of water absorption coefficient for new brick samples in [23] had a value no higher than 38 g/s^0.5^m^2^ and ratio of standard deviation to average water absorption coefficient between 5% and 35%. Bricks obtained from existing buildings and demolition have standard deviation values significantly greater and greater ratio of standard deviation to average water absorption coefficient (Table 3).

**Figure 16:**
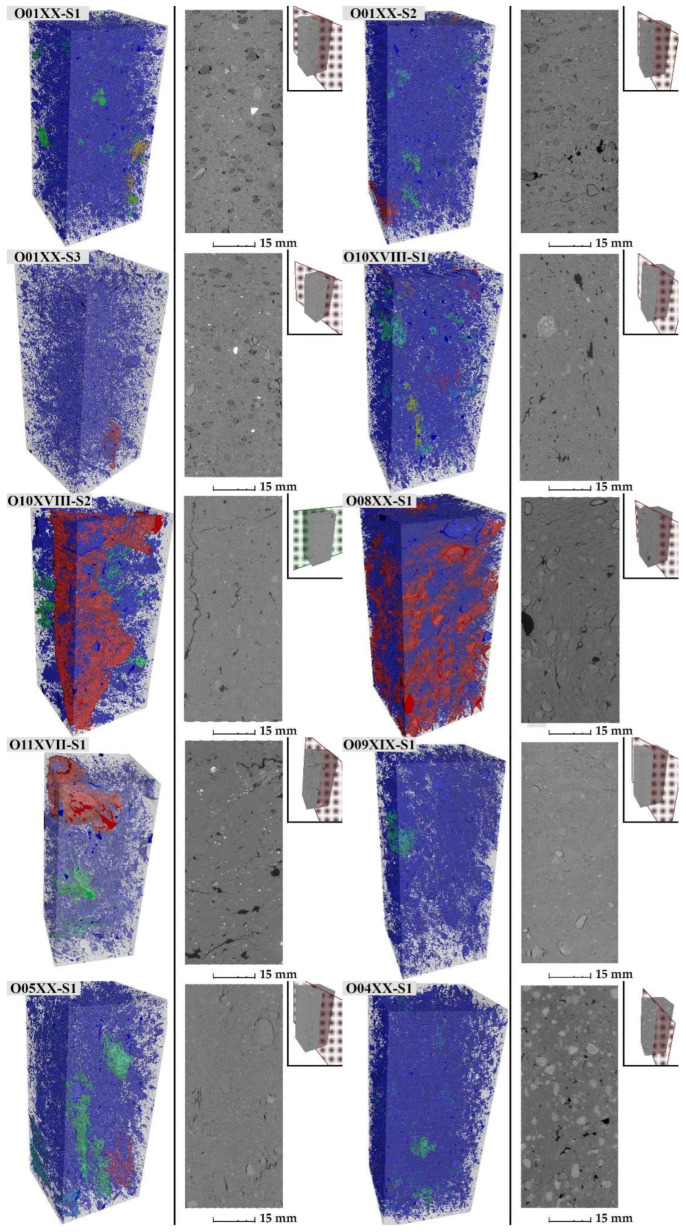
3D view of the pore space from the micro-CT.

**Figure 17:**
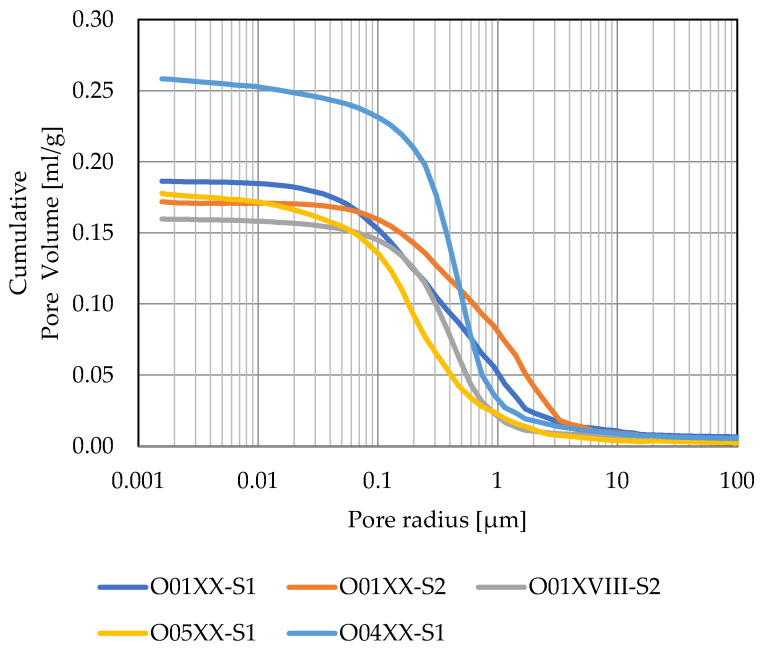
Cumulative pore volume from the MIP test.

From among all the samples, 10 were selected (Table 4) and the pore space was determined using a CT scanner. Samples with similar porosity (approximately 30%) and significant differences in water absorption coefficient were selected from the O01XX, O01XX, O01XX, O10XVIII, O05XX series. Additional samples form O10XVIII, O08XX, O11XVII, O09XIX series were selected due to the fact that they are from earlier centuries than the 20th century. One sample from O04XX series was chosen as a comparison sample with one of the highest porosities. 3D image of pore space is shown in Figure 16. Figure 17 shows cumulative pore volume obtain by MIP method. Pore volume obtained from MIP method (P_MIP_) is greater than those obtained from m-CT (P_CT_). This fact can be explained by a significant contribution of pores smaller than 3 μm in the pore space (Figure 17).

**Figure 18:**
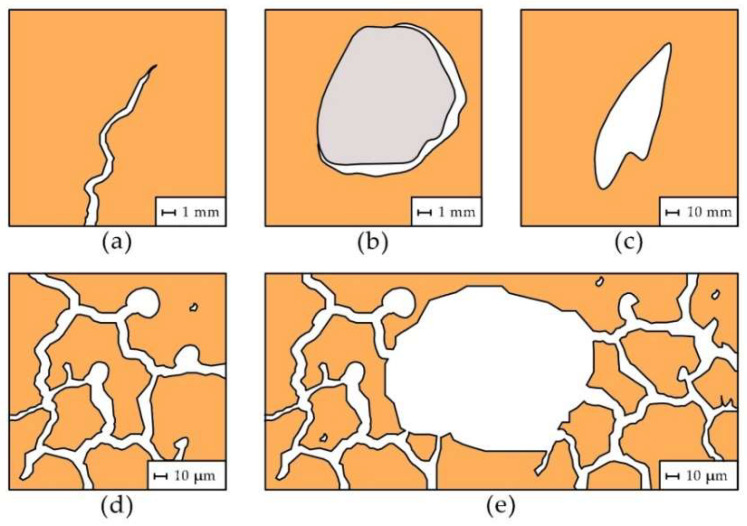
Pore diagram: (**a**)—cracks and fissures, (**b**)—voids around inclusions, (**c**)—voids resulting from under-densification of the material, (**d**)—pores connected to pores of similar diameters, (**e**)—pores that are distributed uniformly in the sample space, connected to pores of much smaller diameters.

Based on the analysis of 3D images for ceramic samples obtained from the micro-CT method and MIP studies, it can be concluded that the pore space consists of (Figure 18):

1. Cracks and fractures;
2. Empty spaces around inclusions;
3. Large spaces resulting from under-densification of the plastic material used for firing (not present in every brick).
4. Pores connected with pores of similar diameters;
5. Pores distributed evenly in the sample, connected to pores of much smaller diameters.

**Figure 19:**
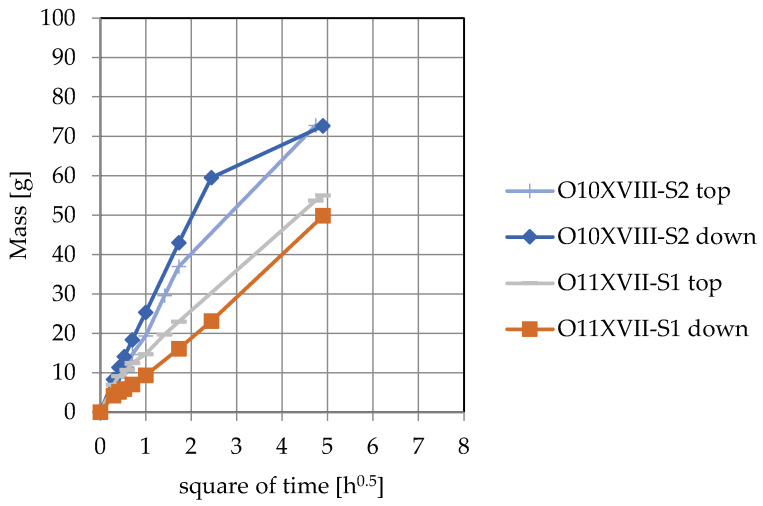
Mass of absorbed water in capillary rise test.

Mass of absorbed water in capillary rise test for chosen samples was shown in the Figure 19. 

In the case of sample O10XVIII-S2, where a crack is visible in Figure 16, when this sample is in contact with the water surface it is noticeable that the value of the water absorption coefficient increased from 219 to 247 g/s^0.5^m^2^. In sample O11XVII, there is a crack around the inclusion grain. When the sample is laid in the capillary rise test with the crack towards the bottom, a decrease in the water absorption coefficient is observed from 115 to 91 g/s^0.5^m^2^. The above observations are in line with the study of Roles et al. [14], in which an increase in the amount of water pulled up was observed when cracks parallel to the direction of movement of the “waterfront” were present in the material. The presence of cracks can cause an increase in the rate of water absorption by the material if the cracks are in contact with the water table. Both spaces around the grains, spaces from under-densification and large pores connected to pores of much smaller diameter will have the effect of reducing the rate of capillary rise. 

## 4. Conclusions

The conducted research, in comparison with data from the literature, indicates that each series of bricks from a given source has its own individual characteristics in terms of compressive strength and the ability of the material to capillary rise of moisture. In the case of samples with similar porosity, but obtained from different sources, there can be up to a six-fold difference in the value of the water absorption coefficient. In addition, imperfections (cracks, voids) present in the bricks can significantly affect the phenomenon of capillary rise. Water absorption coefficient obtain through the conducted experiment are specific and linked to the individual brick.

Good linear correlation between water absorption after 24 h and 7 days and porosity were observed. Correlation coefficient value equals respectively 0.938 and 0.956. Similar pore volume not occupied by water can be observed in samples after 24 h submersion in cold water independently of their porosity, origin and age. Similar volume exists even after 7 days of submersion. This volume is associated by authors as a reason for frost durability observed in the literature.

Taking into account data from Koroth’s doctoral dissertation, it can be deduced that bricks with compressive strength above 80 MPa have a water absorption coefficient of less than 50 g/m^2^s^0.5^.

When m-CT methods were used, pore volume was underestimated due to the resolution. In the case of ceramic bricks, m-CT method is useful for laboratory defects detection. The results of this study highlight the need for easy and cheap method for determination bricks with potentially lower quality, especially in the case of bricks from demolition.

The decrease in compressive strength makes it not possible to reuse demolition bricks.

## Figures and Tables

**Table 1:** Requirements for frost-resistant bricks.

Type of Standard	Compressive Strength		Max. Boiling Absorption 5 h (%)	Max. Saturation Coefficient (-)	Max. Cold Absorption 24 h (%)
	(MPa)	(psi)			
CSA Individual Brick	17.2	-	17.0	0.78	8.0
CSA 5-Brick Average	20.7	-	-	-	-
ASTM Individual Brick	17.2	2500	20.0	0.80	8.0
ASTM 5-Brick Average	20.7	3500	17.0	0.78	-

**Table 2:** Samples used in the study.

Series of Samples	Number of Bricks (Samples)	Age of Samples
N1XXI	17	XXI
N2XXI	4	XXI
O01XX	5	XX
O02XX	2	XX
O03XX	5	XX
O04XX	5	XX
O05XX	4	XX
O06XX	10	XX
O07XX	9	XX
O08XX	2	XX
O09XIX	2	XIX
O10XVIII	2	XVIII
O11XVII	2	XVII

**Table 3:** Water absorption coefficient average value and standard deviation for selected series.

Series	Average Water Absorption Coefficient (g/s^0.5^m^2^)	Standard Deviation (g/s^0.5^m^2^)
N1XXI	172.2	40.86
O01XX	134.5	57.03
O04XX	316.0	126.05
O06XX	128.7	42.64
O07XX	380.45	70.96

**Table 4:** Results of water absorption coefficient and porosity determined by hydrostatic method, MIP, micro-CT.

Sample	C_1_—Water Absorption Coefficient (g/s^0.5^m^2^)	Porosity—P_HYD_ (%)	Porosity—P_MIP_ (%)	Porosity—P_CT_ (%)
O01XX-S1	156.32	32.05	32.78	2.31
O01XX-S2	301.52	31.96	31.13	2.38
O01XX-S3	171.63	32.65	X	1.41
O10XVIII-S1	301.34	28.28	X	2.84
O10XVIII-S2	231.92	31.28	29.29	3.09
O08XX-S1	87.80	19.26	X	7.88
O11XVII-S1	94.95	27.79	X	3.34
O09XIX-S1	55.96	16.91	X	1.79
O05XX-S1	81.33	30.50	31.52	2.07
O04XX-S1	258.65	40.70	40.62	3.33

X—MIP study not performed, P_HYD_—porosity determined by the hydrostatic method, P_MIP_—porosity determined by the MIP method, P_CT_ porosity determined by microcomputed tomography.

## Data Availability

Not applicable.
